# Healthcare Experiences of Underrepresented Lesbian and Bisexual Women: A Focus Group Qualitative Study

**DOI:** 10.1089/heq.2017.0041

**Published:** 2018-07-01

**Authors:** Sue LaVaccare, Allison L. Diamant, Julie Friedman, Karen T. Singh, Jessica A. Baker, Tayler A. Rodriguez, Susan R. Cohen, Farina Y. Dary, Janet Pregler

**Affiliations:** ^1^ Los Angeles County Lesbian and Bisexual Women's Health Collaborative, Los Angeles, California.; ^2^ Department of Internal Medicine, David Geffen School of Medicine at UCLA, Los Angeles, California.; ^3^ Iris Cantor-UCLA Women's Health Education and Research Center, Los Angeles, California.; ^4^ Department of Health Sciences, California State University, Northridge, Northridge, California.; ^5^ Project RENEW, Pathways by Molina, Costa Mesa, California.; ^6^ Iris Cantor-UCLA Women's Health Center, Los Angeles, California.

**Keywords:** sexual orientation, healthcare environment, disclosure

## Abstract

**Purpose:** To understand the complex healthcare experiences of women identifying as lesbian or bisexual. who are also women of color, veterans, and/or 65 years of age and older.

**Methods:** Inclusion criteria were age 25 or older, Los Angeles County resident, self-identification as a lesbian or bisexual woman, and as an African American, Latina, Asian-Pacific Islander, and/or a veteran. For the age 65 years and older group, participants were eligible regardless of their veteran status or race/ethnicity. Five focus groups were conducted (*n*=35) and the same questions were asked addressing their comfort interacting with healthcare providers, the provider knowing their sexual orientation, characteristics of a perfect provider, and barriers to care. Structured qualitative analyses were performed.

**Results:** Participants identified concerns that providers often hold to heterosexual cultural norms. Participants varied on preferences for providers of the same race/ethnicity as themselves. Lesbians who are 65 years and older identified legal barriers as major concerns. All groups identified incorrect provider assumptions about sexual orientation and sexual practices as frequently compromising their care. Participants supported the idea of certification for providers skilled in lesbian, gay, bisexual, transgender, and queer (LGBTQ) health, but expressed skepticism that such programs would necessarily result in better care.

**Conclusion:** Healthcare provider trainings need to address the specific concerns and experiences of underrepresented lesbian and bisexual women. Healthcare environments must be transformed to effectively address their needs. More research is needed on the separate healthcare experiences of specific marginalized populations related to their sexual orientation and/or gender identity.

## Introduction

The unique healthcare experiences of lesbian and bisexual (LB) women are overshadowed by research focused primarily on heterosexual females and homosexual males.^1^ However, existing LB research focuses predominantly on white, middle-class women and indicates that they face significant health disparities as revealed in a 2011 Institute of Medicine report.^2^ The report demonstrated that LB women seek healthcare less frequently, are less likely to receive preventive healthcare such as Pap tests and mammograms, and have lower adherence rates for prescribed treatment protocols compared to their heterosexual counterparts.^2^ LB women not disclosing are less likely to seek healthcare.^3–9^ Providers compromise care for LB women when they do not create safe and encouraging environments for their patients to disclose their sexual orientations.^4,5,10–15^ Rates of nondisclosure to healthcare providers are 32.6% for bisexual women and 12.9% for lesbians.^7^ Medical settings welcoming disclosure increase the likelihood of LB women to seek and obtain necessary and appropriate healthcare.^16^

Presently, the healthcare experiences of LB women who are older, of color, and are veterans remain poorly understood. Studies of LB women from these marginalized populations demonstrate additional significant disparities. One study found that LB veterans avoid accessing medical services from the Veterans Administration.^17^ There is a higher prevalence of chronic medical conditions and a lower rate of preventative care utilization among LB women of color than among heterosexual women.^8,9,16,18–23^ Among older LGBT adults, 21% have not revealed their sexual orientation to their primary healthcare provider.^24^ Lesbian women of color and those who are immigrants report higher rates of nondisclosure compared to white women and those who are U.S. born.^7^ Regardless of sexual orientation, African Americans, Asian Americans, and Hispanics are more likely to experience disrespect during their healthcare experiences in comparison to whites, which may contribute to these high nondisclosure rates.^25^ In addition, internalized homophobia and the lack of connection to others identifying as lesbian, gay, bisexual, transgender, and queer (LGBTQ) within their ethnic communities may hinder disclosure.^7^

The aim of this 2012 focus group study was for the Los Angeles County Lesbian and Bisexual Women's Health Collaborative (LBWHC) to contribute a multidimensional and culturally diverse perspective on the healthcare experiences of LB women underrepresented in research. The mission of LBWHC is to address the health disparities of LB women facing added marginalization. The Iris Cantor-UCLA Women's Health Center spearheads LBWHC, whose members represent healthcare organizations, government agencies, community organizations, and academic institutions.

## Methods

LBWHC selected the five focus groups to represent the “multiple minority” of being a woman, identifying as a member of a sexual minority, and belonging to an underrepresented group in healthcare research. Participants were eligible if they met the following criteria: (1) were a lesbian or bisexual woman, (2) were aged 25 or older, and (3) were a veteran, 65 years of age or older, or African American, Asian-Pacific Islander (API), or Latina. African Americans, APIs, and Latinas were selected for inclusion as ethnic minorities. LB veterans and women aged 65+ were multiethnic and selected to understand other populations facing discrimination. A distinct focus group was conducted for each of these five underrepresented groups. Each participant provided verbal consent and received a $15 gift card. The UCLA Institutional Review Board approved the study.

Los Angeles County provided a large metropolitan area to examine the five groups separately, while also analyzing their commonalities and differences. Recruitment messages were disseminated at the Los Angeles LGBT Center, the Asian Pacific American Legal Center, a nightclub frequented by African American LB women, LGBTQ events, and through personal contacts.

Each focus group included 3 to 13 participants and was 90-min long. At the beginning of each focus group, participants completed an anonymous sociodemographic pen and paper survey. Focus group and survey questions were developed with assistance from UCLA research and clinical faculty experienced in LGBTQ issues, and explored a variety of healthcare-related topics (Table 1). One researcher with experience leading LGBTQ focus groups facilitated all groups. In addition, a co-facilitator from each underrepresented community provided further expertise in group facilitation. All focus groups were conducted in English, audio recorded, and transcribed.

**Table 1.** **Questions Asked to Focus Group Participants**

Focus Group Questions
1. Do you feel comfortable discussing all aspects of your health with your provider, or do you feel uncomfortable discussing certain aspects?
2. In your opinion, how important is it that your healthcare provider knows of your sexual orientation?
3. If you could describe the perfect healthcare provider, what qualities would you like him or her to possess and do you have a preference if the provider is male or female?
4. What barriers limit access to healthcare for lesbians and bisexual women?

The analytic approach was congruent with qualitative research models where categories of data emerge.^26^ An initial thematic analysis was conducted by four researchers. Each researcher was given the five focus group transcripts for independent review and coding. Coded data originally generated nine thematic categories, which were collapsed into five major themes and became the basis for the codebook. Themes included the following: the impact of race and ethnicity; disclosure of sexual orientation to healthcare providers; legal protection; healthcare environment; and provider training and certification in LGBTQ health (Table 2). Two subthemes emerged, specifically, patient-provider relationships regarding disclosure and end-of-life planning under legal protection. The focus group transcripts were reread iteratively, the participant data were reanalyzed in light of the emerging themes, and the quotations best reflecting the opinions of participants were selected.

**Table 2.** **Number of Participant Responses to Themes and Subthemes**

Focus group	Total number of participants	Theme 1: disclosure of sexual orientation to healthcare providers	Theme 1a: patient-provider relationships	Theme 2: provider training and certification in LGBTQ health	Theme 3: healthcare environment	Theme 4: impact of race and ethnicity	Theme 5: legal protection	Theme 5a: end-of-life planning
African American	5	1	5	5	4	1	0	0
Asian-Pacific Islander	7	6	7	7	4	6	0	0
Latina	7	2	5	6	4	2	0	0
Veteran	3	0	3	3	2	0	0	0
65+	13	0	6	1	3	0	2	3

Total number of explicit responses to each theme.

LGBTQ, lesbian, gay, bisexual, transgender, and queer.

## Results

### Sociodemographics

Thirty-five participants were recruited for this study. The sample size in each of the focus groups was as follows: 5 in the African American group, 7 in the API group, 7 in the Latina group, 3 in the veterans group, and 13 in the 65+ group. Sociodemographic results for the entire sample are found in Table 3. Eighty percent (*n*=28) of participants identified as lesbian. This included 100% (*n*=5) of African American participants, 57% (*n*=4) of participants each from the API and Latina groups, 100% (*n*=3) of veterans, and 92% (*n*=12) of participants from the 65+ group (Table 3). The remaining 20% (*n*=7) of participants identified as bisexual, including 43% (*n*=3) of the API group, 43% (*n*=3) of the Latina group, and 8% (*n*=1) of the 65+ group.

**Table 3.** **Demographic Characteristics by Focus Group**

	Full sample count nf=35	%	African American count naa=5	%	Asian-Pacific Islander count nai=7	%	Latina count nl=7	%	Veterans count nv=3	%	65+ count n+=13	%
Race/ethnicity												
Caucasian	11	31.42	0	—	0	—	0	—	1	33.00	10	77.00
African American	7	20.00	5	100.00	0	—	0	—	2	67.00	0	—
Asian-Pacific Islander	7	20.00	0	—	7	100.00	0	—	0	—	0	—
Latina	8	22.80	0	—	0	—	7	100.00	0	—	1	8.00
Nonrace	1	2.86	0	—	0	—	0	—	0	—	1	8.00
Other	1	2.86	0	—	0	—	0	—	0	—	1	8.00
Sexual orientation												
Lesbian	28	80.00	5	100.00	4	57.14	4	57.14	3	100.00	12	92.31
Bisexual	7	20.00	0	—	3	42.86	3	42.86	0	—	1	7.69
Age range												
18–29	8	22.86	0	—	5	71.43	3	42.86	0	—	0	—
30–39	7	20.00	1	20.00	1	14.29	3	42.86	2	66.67	0	—
40–49	3	8.57	1	20.00	1	14.29	1	14.29	0	—	0	—
50–59	2	5.71	2	40.00	0	—	0	—	0	—	0	—
60–64	1	2.86	1	20.00	0	—	0	—	0	—	0	—
65+	14	40.00	0	—	0	—	0	—	1	33.33	13	100.00
Employment												
Full time	12	34.29	2	40.00	4	57.14	5	71.43	1	33.33	0	—
Part time	4	11.43	1	20.00	1	14.29	1	14.29	0	—	1	7.69
Unemployed	4	11.43	1	20.00	2	28.57	0	—	1	33.33	0	—
Retired	13	37.14	0	—	0	—	0	—	1	33.33	12	93.31
Other	2	5.71	1	20.00	0	—	1	14.29	0	—	0	—
Doctor visit in the past 6 months												
Yes	29	82.86	1	20.00	6	85.71	7	100.00	2	66.67	13	100.00
Relationship status												
Not in a relationship	30	85.71	5	100.00	7	100.00	7	100.00	1	33.33	10	76.92
In a relationship	5	14.29	0	—	0	—	0	—	2	66.67	3	23.08

Note: Percentages are estimated using the available data.

### Theme 1: disclosure of sexual orientation to healthcare providers

All focus groups expressed the importance of healthcare providers knowing their patients' sexual orientations, either by patients stating it verbally or acknowledging it as a choice on an intake form. Participants' individual decisions to disclose depended on their level of comfort with their providers, as well as other factors specific to certain groups. For African American and Latina participants, their decisions to disclose were influenced by heteronormative assumptions made by their providers regarding their sexual behaviors and orientations. In both groups, some participants disclosed their sexual orientations to avoid assumptions from being made. Others expressed discomfort in disclosing their sexual orientations because assumptions were already made by healthcare providers. An African American participant explained, “it is often assumed that you are heterosexual and that is uncomfortable… it is important that they know, but I don't volunteer that information…”

In the API and 65+ groups, participants also shared experiences in which providers made incorrect assumptions about their sexual behaviors and/or sexual orientations; however, their decisions to disclose their sexual orientations were influenced by different factors. For API participants, concordant race/ethnicity with their healthcare providers was also seen as a possible barrier. Some participants expressed hesitance to disclose to a provider of the same ethnicity on the basis that providers might know their families or be uncomfortable with their sexual orientations. One API participant highlighted this when she said, “I switched doctors because my primary care physician was Korean and even though she's second generation… I still felt like I couldn't come out because I had no idea how she stood on that.” Another API participant was hesitant to disclose because of concerns that her sexual orientation would not remain confidential within her ethnic community.

A prevalent reason for disclosure among the 65+ group and one veteran was to have their partner included in care and decision-making. Despite the negative experiences aforementioned, some 65+ participants reported positive interactions with physicians after disclosing their sexual orientations. For example, a 65+ participant shared that when she and her partner were discussing a test the physician was trying to convince her to get, the physician said, “you know, she loves you more than you love yourself, maybe you ought to listen to her.”

### Theme 2: provider training and certification in LGBTQ health

All groups felt that providers need specific training to better serve the LGBTQ community. The Latina participants indicated that trained providers would improve their healthcare visits. Two API participants stated that all medical providers should have mandatory training on issues affecting the LGBTQ community; another two API participants expressed that the training should be continuously updated. A 65+ participant stated that providers need extra training to better understand and care for LGBTQ patients. Participants from the African American, Latina, and veteran groups expressed preferences for providers with training certification in LGBTQ health over providers without certification.

With the exception of the 65+ group, all other groups expressed some skepticism toward provider certification in LGBTQ health. Questions were raised about the content of the training curriculum and what a certification in LGBTQ health would denote. Participants expressed concern that certification would not be indicative of the provider's sincerity in caring for the LGBTQ community. An African American and veteran participant explained that even if a provider is certified, positive personal interactions with them are more important. API participants expressed that despite training in LGBTQ health, providers would still need racial/ethnic awareness to provide comprehensive patient care.

### Theme 3: healthcare environment

In all focus groups, except for the 65+ group, comments and suggestions were made to improve the welcoming nature of healthcare facilities, as a few participants encountered sites that were unreceptive to their sexual orientations. For example, an African American participant recalled her discomfort upon walking into a waiting room geared toward heteronormative family planning. Participants expressed a desire to see visual cues signifying that the healthcare facility is welcoming of the LGBTQ population and suggested incorporating images of same-sex couples in posters, brochures, and magazines displayed in medical offices. Two participants in the Latina group suggested that posters and pamphlets including lesbian and gay couples and health information relevant to the LGBTQ population should be more readily available. Similarly, one participant in the African American group felt that a welcoming environment might include a rainbow flag in the waiting room as well as a friendly and positive reception by healthcare staff and providers. Furthermore, one API participant suggested the inclusion of queer magazines in waiting rooms, and another suggested that staff wear a pin to signal that they are LGBTQ friendly.

All focus groups favored intake forms where patients could indicate their sexual orientations. A Latina participant shared, “I like the idea of the form…it helps to normalize the experience for everybody whether you're comfortable or not and helps break that stigma.” One African American participant felt that a form could help providers “steer their questions” to be inclusive of the patient's sexual orientation.

### Theme 4: impact of race and ethnicity

African American, API, and Latina focus group participants prefer a provider of the same race/ethnicity. The veteran and 65+ groups did not mention race/ethnicity as a factor influencing their provider choice. An African American participant stressed the importance of having a provider of the same race, even if they did not share the same sexual orientation, because it allows her to identify with them and build a “kinship” or stronger patient-provider relationship. For API and Latina participants, accessing healthcare services that are receptive to both their race/ethnicity and sexual orientation was important because of the cultural stigma that exists around being an LB woman. A Latina participant explained these barriers by stating, “I think that coming from the Latina community where it's still very much looked down upon to be gay, and you're kind of a first-generation lesbian, in most cases you really have to seek out medical services and if there is a way to take that into consideration…that would help a lot because there's already this discomfort in looking for and accessing services that are LGBT sensitive.” In addition, aspects of language were recurrent issues among API participants. One participant expressed a need for more providers who are bilingual/multilingual. Two other participants expressed a need for LGBTQ terminology specific to their ethnic languages.

### Theme 5: legal protection

The 65+ focus group was the only group where legal protection was of significant concern. One participant stressed the importance of disclosing her sexual orientation to avoid situations in which her same-sex partner would be denied visitation rights or access to health information. The participant recounted going to a new physician and completing the medical forms, saying “… I put my partner's name down and her phone number and… I was really clear about it—that I'm a lesbian and she's a lesbian and we are together.” Another participant explained that after her partner passed away, she lost her health insurance through her partner's work because their relationship was not legally recognized.

Three other 65+ participants spoke about the importance of advance care directives to guarantee involvement in the decision-making process of their partner's care. However, one participant shared that even though her partner had an advance directive on file, it was overlooked and she had to proactively make providers aware.

## Discussion

Our LB qualitative study indicates that women of color, veterans, and those who are 65+ face multiple barriers when seeking quality healthcare. The results build upon previous work suggesting that changes in healthcare settings can promote inclusive care.^5,27–33^ A reference guide of barriers and solutions to inclusive care can be found in Table 4. Participants discussed the impact of disclosure of sexual orientation, provider training, welcoming healthcare environments, and race/ethnicity on their healthcare experiences.

**Table 4.** **Barriers and Solutions to Quality Healthcare for Lesbians and Bisexual Women**

Participant-identified barriers	Potential solutions
Lack of disclosure of sexual orientation	Provider level: Encourage training in interpersonal skills for providers by providing education on lesbian and bisexual women's issues specifically.
	Clinic/Systems level: Make asking about sexual orientation “routine” during intake questionnaires; configure EHR systems so that for patients who choose to disclose sexual orientation, the information is immediately and appropriately available.
Gaps in provider knowledge	Provider level: Increased provider training.
	Systems level: Include issues around lesbian and bisexual women's health in quality improvement initiatives.
Poor provider interpersonal skills related to lesbian and bisexual women's issues	Provider level: Encourage training in interpersonal skills for providers with a focus on lesbian and bisexual woman-specific issues.
	Systems level: Collect data on patient satisfaction with interactions that are stratified by sexual orientation and use to feed back to individual clinicians, and for quality improvement.
Noninclusive office/clinic/hospital environments	Clinic/Systems level: Provide training to staff on lesbian and bisexual women' issues.
	This includes all staff who interact with patients on any issue (e.g., those who make appointments, collect information, provide financial counseling/help with billing, etc.); make questionnaires and EHR relevant and pertinent to lesbian and bisexual women. Ensure artwork, pamphlets, magazines, etc. are inclusive of lesbian and bisexual women.
Lack of lesbian and bisexual woman-friendly and woman-competent providers with relevant cultural and linguistic skills to serve minority communities	Provider level: Provider-specific education.
	Systems level: Encourage directories or referral services to link patients with providers who identify as providing these services. Collect quality and patient satisfaction data to provide objective information on providers who self-identify as lesbian and bisexual woman friendly/competent.
Lack of policies or lack of enforcement of polices about hospital visitation and medical decision-making rights for lesbian couples	Provider level: Provider education specific to these issues.
	Systems level: Encourage participation of healthcare organizations in initiatives to improve policies and processes (e.g., Health Equity Index program of the Human Rights Campaign); support system-wide efforts (including her-based initiatives) to encourage identification and appropriate documentation of surrogate decision makers for all patients, including those who identify as lesbian or bisexual women.

EHR, electronic health record.

Our findings on LB women's healthcare experiences, particularly on the barriers that exist for underrepresented LB women when disclosing their sexual orientations, can be understood through the Personal Risking Theory (PRT). This theory, developed by Hitchcock and Wilson, suggests that lesbians go through two phases when considering disclosure: an anticipatory phase that occurs before entering the healthcare facility and an interactional phase that occurs upon entering the healthcare facility.^12,34^ During the anticipatory phase, lesbians may imagine the conversations they will have with staff and/or investigate whether or not providers are LGBTQ friendly.^34^ During the interactional phase, lesbians may look for LGBTQ materials in the waiting room, blank spaces instead of boxes on intake forms inquiring about sexual orientation, and/or observe physicians to consider how they might react to disclosure.^34^

This study supports the PRT as it applies to lesbians and suggests that bisexual women be considered in applications of the theory. This study also identifies factors impacting the anticipatory and interactional phases of disclosure. Race/ethnicity concordance, legal protection for LB women, and provider certification in LGBTQ health appear to influence the anticipatory phase. The race/ethnicity of the provider can either encourage or dissuade LB women from disclosing, particularly API and Latina women. Legal protection often compels 65+ participants to disclose and provider certification may encourage disclosure among LB women. Patient-provider relationships and the surrounding healthcare environment were found to affect the interactional phase of disclosure. Similar to prior studies, heteronormative presumptions made during visits were found to negatively influence disclosure.^4,7,8,35,36^ In addition, creating LGBTQ-friendly environments with affirming cues was found to likely encourage patient disclosure.^12,37^ Intake forms with boxes to indicate sexual orientation also appeared to be a desirable option for disclosure.

All groups in this study endorsed training providers in LGBTQ health, which is recommended in prior studies.^38,39^ Similarly, as seen in research on heterosexual persons of color, our research shows that LB women of color also desired providers who were of the same race/ethnicity.^40–43^ API participants wanted bilingual or multilingual physicians, which was consistent with other studies that found that a shared ethnic identity was important for patients who had concerns about communicating in their native language.^40,41^ Overall, participants wanted racial/ethnic concordant providers who are also LGBTQ friendly. Previous studies demonstrate that LGBTQ patients who felt that they could trust their provider were more likely to disclose their sexual orientations, which our study supports.^3,4,8,12,35^

### Study Limitations

This study is limited in its ability to broadly represent the myriad concerns of LB women. Focus group recruitment, especially for the veteran group, was a challenge. In addition, 77% of participants in the 65+ group were Caucasian, which potentially skewed the results. Selection bias was present as some participants knew the cohort facilitator or other participants. Furthermore, the few questions asked were narrow in scope, which contributed to tangential discussions diluting the data. This study did not differentiate between LB women, although they have different health concerns, rates of disclosure, interactions with providers, and experiences in accessing healthcare.^44–47^

## Conclusion

This study highlights disparities related to disclosure, inclusivity, and patient-provider interactions among subgroups of LB women. Training health professionals in LB care and creating environments reflective of the whole patient population, with respect to factors such as race/ethnicity and age, are required for inclusive care. For LB women of color, receiving care in their native languages, where providers use terminology that is respectful of their sexual orientations is essential. Recruiting providers of color who identify as LGBTQ themselves, or are trained in LGBTQ health, is critical to improve the healthcare experiences for underrepresented LB women.

Studying groups of underrepresented LB women separately, rather than as one category of marginalized women, is important for uncovering their specific challenges. Since this study was conducted in 2012, LGBTQ persons have more accepted choices in the terminology used when describing their sexual orientations and gender identities. Research is needed on the barriers in healthcare faced by queer and transgender individuals identifying as lesbian, bisexual, or queer. Further research is also needed to explore the healthcare experiences and needs of LB immigrant women of color. Including these and other subgroups in research can improve the healthcare experiences and health outcomes for all LB women and queer-identifying individuals. Although these marginalized populations are small, their needs are significant and should not be overlooked.

## Abbreviations Used

API: Asian-Pacific Islander
EHR: electronic health record
LB: lesbian and bisexual
LBWHC: Lesbian and Bisexual Women's Health Collaborative
LGBTQ: lesbian, gay, bisexual, transgender, and queer
PRT: Personal Risking Theory
